# Dexamethasone-Enhanced Continuous Online Microdialysis for Neuromonitoring of O_2_ after Brain Injury

**DOI:** 10.1021/acschemneuro.2c00703

**Published:** 2023-06-27

**Authors:** Elaine M. Robbins, Andrea S. Jaquins-Gerstl, David O. Okonkwo, Martyn G. Boutelle, Adrian C. Michael

**Affiliations:** † Department of Chemistry, University of Pittsburgh, 219 Parkman Avenue, Pittsburgh, Pennsylvania 15260, United States; ‡ Department of Neurological Surgery, University of Pittsburgh School of Medicine, Pittsburgh, Pennsylvania 15213, United States; § Department of Bioengineering, Imperial College London, London SW7 2AZ, United Kingdom

**Keywords:** Microdialysis, dexamethasone, oxygen, spreading depolarization, traumatic brain injury, controlled cortical impact

## Abstract

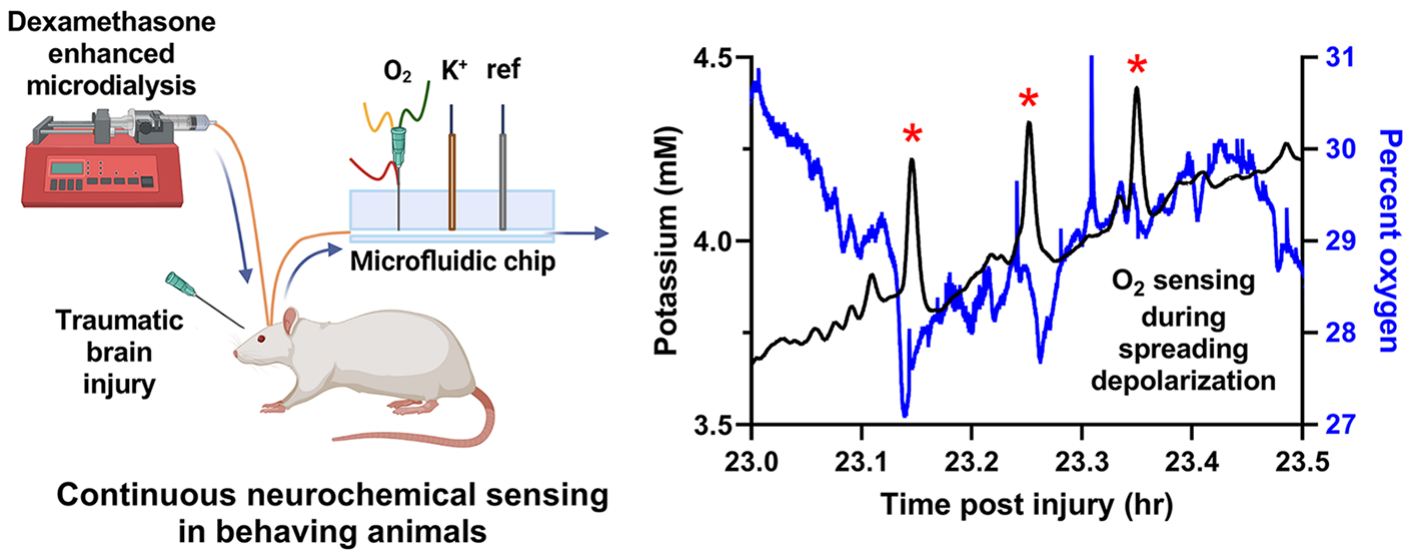

Traumatic brain injury (TBI) is a major public health crisis in many regions of the world. Severe TBI may cause a primary brain lesion with a surrounding penumbra of tissue that is vulnerable to secondary injury. Secondary injury presents as progressive expansion of the lesion, possibly leading to severe disability, a persistent vegetive state, or death. Real time neuromonitoring to detect and monitor secondary injury is urgently needed. Dexamethasone-enhanced continuous online microdialysis (Dex-enhanced coMD) is an emerging paradigm for chronic neuromonitoring after brain injury. The present study employed Dex-enhanced coMD to monitor brain K^+^ and O_2_ during manually induced spreading depolarization in the cortex of anesthetized rats and after controlled cortical impact, a widely used rodent model of TBI, in behaving rats. Consistent with prior reports on glucose, O_2_ exhibited a variety of responses to spreading depolarization and a prolonged, essentially permanent decline in the days after controlled cortical impact. These findings confirm that Dex-enhanced coMD delivers valuable information regarding the impact of spreading depolarization and controlled cortical impact on O_2_ levels in the rat cortex.

## Introduction

Traumatic brain injury (TBI) due to vehicle and industrial accidents, sports injuries, military action, etc. is a major public health crisis.^1,2^ TBI may produce a primary brain lesion surrounded by a penumbra of tissue vulnerable to secondary injury.^3−5^ This creates an urgent need for technology to detect and monitor secondary injury. Prior studies affirm that neuromonitoring by intracranial microdialysis,^2−7^ which is approved for clinical use in several countries, is a foundation for such technology.

Several mechanisms have been implicated in secondary brain injury, including ischemia, elevated intracranial pressure, seizure, etc.^8−12^ Recently, however, spreading depolarization (SD) has become a central focus because SD is detected in approximately 60% of monitored TBI patients and because a correlation has been established between SD and patient outcome.^13,14^ SD is a pathological disturbance of homeostasis that spreads as a wave across the cortex (a “brain tsunami”^15^): the disruption of transmembrane ion gradients results in mass cellular depolarization.^16,17^ Following SD, the tissue consumes vast amounts of energy during the restoration of homeostasis: if the energy demand exceeds the supply, metabolic crisis leading to tissue death ensues.^18^ Thus, a goal of neuromonitoring is to detect metabolic crisis in real-time.

Microdialysis is a well-established tool for intracranial neurochemical monitoring.^19−21^ Dexamethasone-enhanced^22−26^ continuous online microdialysis^27−31^ (Dex-enhanced coMD) is an emerging paradigm for long-term neuromonitoring after brain injury.^26,32,33^ Retrodialysis with dexamethasone mitigates the foreign body response to probe insertion that otherwise degrades the functionality of microdialysis over time. The adoption of continuously operating sensors for analysis of the dialysate stream delivers the high temporal resolution necessary to monitor the chemical dynamics associated with SD.

The focus of the present study is the real-time monitoring of interstitial oxygen in the rat cortex with Dex-enhanced coMD. The availability of O_2_, alongside glucose, is critical to meeting the energy requirements for the restoration of homeostasis following SD. In acute experiments, we manually induced SD by a pin-prick to the cortical surface of anesthetized rats. In chronic experiments (defined here as 7 days following probe insertion), we monitored SD after controlled cortical impact (CCI), a widely used rodent model of TBI.^34−36^ The O_2_ results reported herein show similarities to the glucose data reported recently by Robbins et al.,^32^ in that SD induces a variety of O_2_ responses. Moreover, dialysate O_2_ levels exhibit a prolonged, essentially permanent, decline to very low concentrations in the days after CCI. Collectively, our findings support the idea that 7-day Dex-enhanced coMD offers valuable information regarding the impact of SD and CCI on O_2_ levels in injured brain tissue.

## Results and Disucssion

### Experimental Design

**Figure 1:**
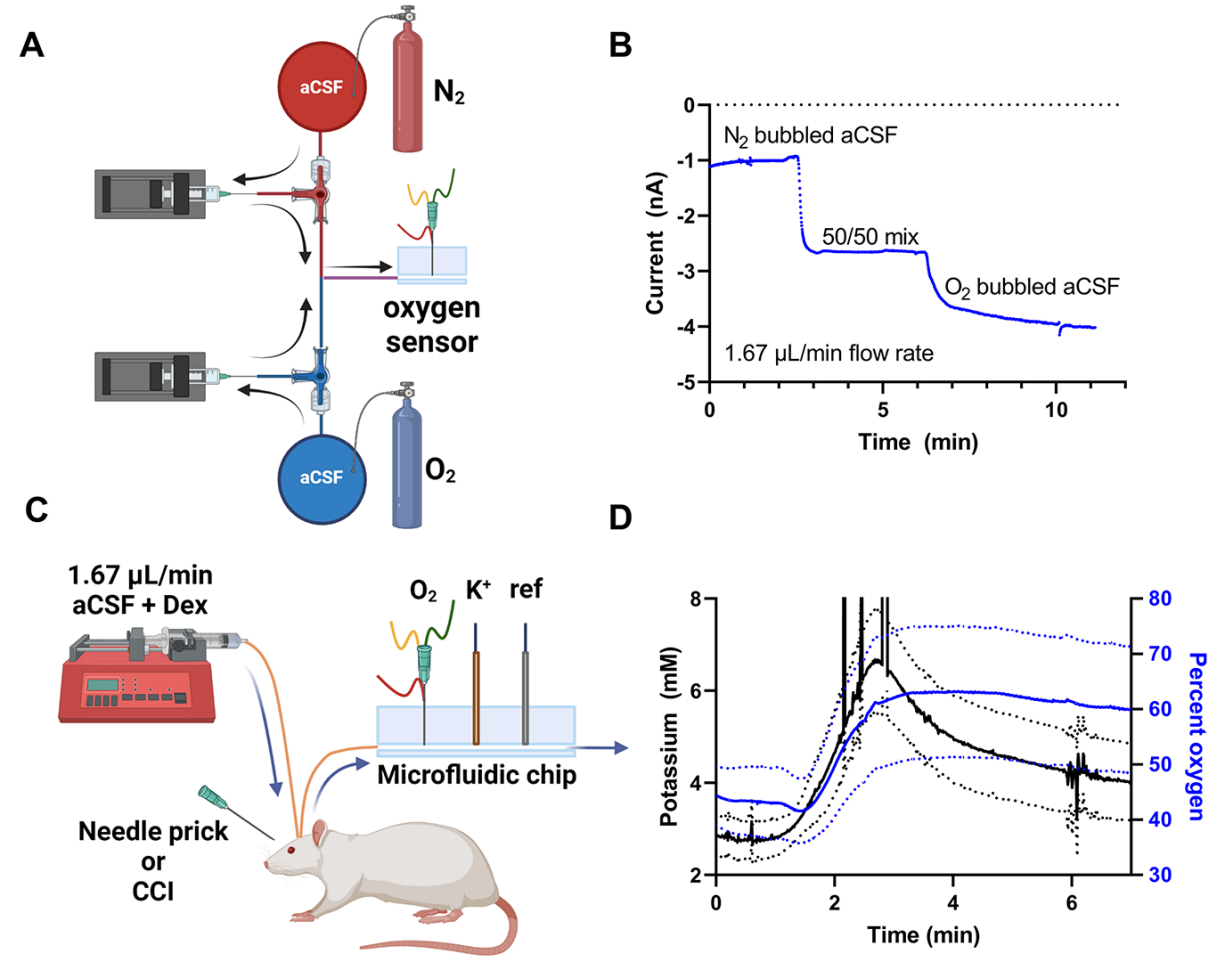
Schematics of the Dex-enhanced coMD system for O_2_. (A) Setup for O_2_ calibration. (B) Representative O_2_ calibration. (C) Setup for monitoring O_2_ and K^+^ in the rat cortex. (D) K^+^ (black, left axis) and O_2_ (blue, right axis) responses to probe insertion (solid lines are the mean, dotted lines show ±SEM).

Figure 1 is a schematic of the Dex-enhanced coMD system for monitoring O_2_. The O_2_ sensors, 50 μm diameter Pt disk electrodes, were mounted in a 3D-printed electrochemical detector and calibrated prior to being connected to the microdialysis probes. We calibrated the O_2_ electrodes by connecting the electrochemical detector with fused silica tubing to a pair of pumps fitted with gastight syringes that delivered varying combinations of aCSF equilibrated with N_2_ or O_2_ gas (Figure 1A). Fused silica capillary tubing was selected for this application because it is impermeable to O_2_ and preserves the O_2_ concentration in the sample stream. Figure 1B shows an O_2_ calibration: we report O_2_ data as “percent O_2_”, defining aCSF equilibrated with N_2_ as 0% and aCSF equilibrated with O_2_ as 100%. The same pumps were used to calibrate the K^+^ μISE. Microdialysis probes were inserted into the rat cortex and perfused at 1.67 μL/min with air-equilibrated artificial cerebrospinal fluid (aCSF) containing Dex (Figure 1C, see Methods for the details of Dex retrodialysis). The outlet of the microdialysis probe was connected via fused silica capillary tubing to a 3D printed electrochemical detector equipped for the amperometric detection of O_2_ and the potentiometric detection of K^+^. We induced SD either by means of a manual pin-prick to the brain surface (acute experiments) or by CCI (7-day chronic experiments).

### Microdialysis Probe Insertion

We monitored the dialysate of *n* = 11 anesthetized rats during the insertion of microdialysis probes into the cortex (Figure 1D). Figure 1D shows the average (±SEM) of the dialysate K^+^ and O_2_ responses to probe insertion on a time axis corrected to account for the transit time of dialysate through the probe outlet line (determined for each probe individually). As reported before,^26^ insertion of a microdialysis probe induces a transient rise in K^+^ from the initial concentration of 2.7 mM in the aCSF present in the probe prior to insertion. Upon insertion, dialysate O_2_ levels rose from near 40%, corresponding to aCSF equilibrated with ambient air, to a maximum level in brain tissue near 60% and then slowly declined. However, dialysate O_2_ levels did not exhibit a transient response to the insertion.

### Acute Measurements: Manually Evoked SD

**Figure 2:**
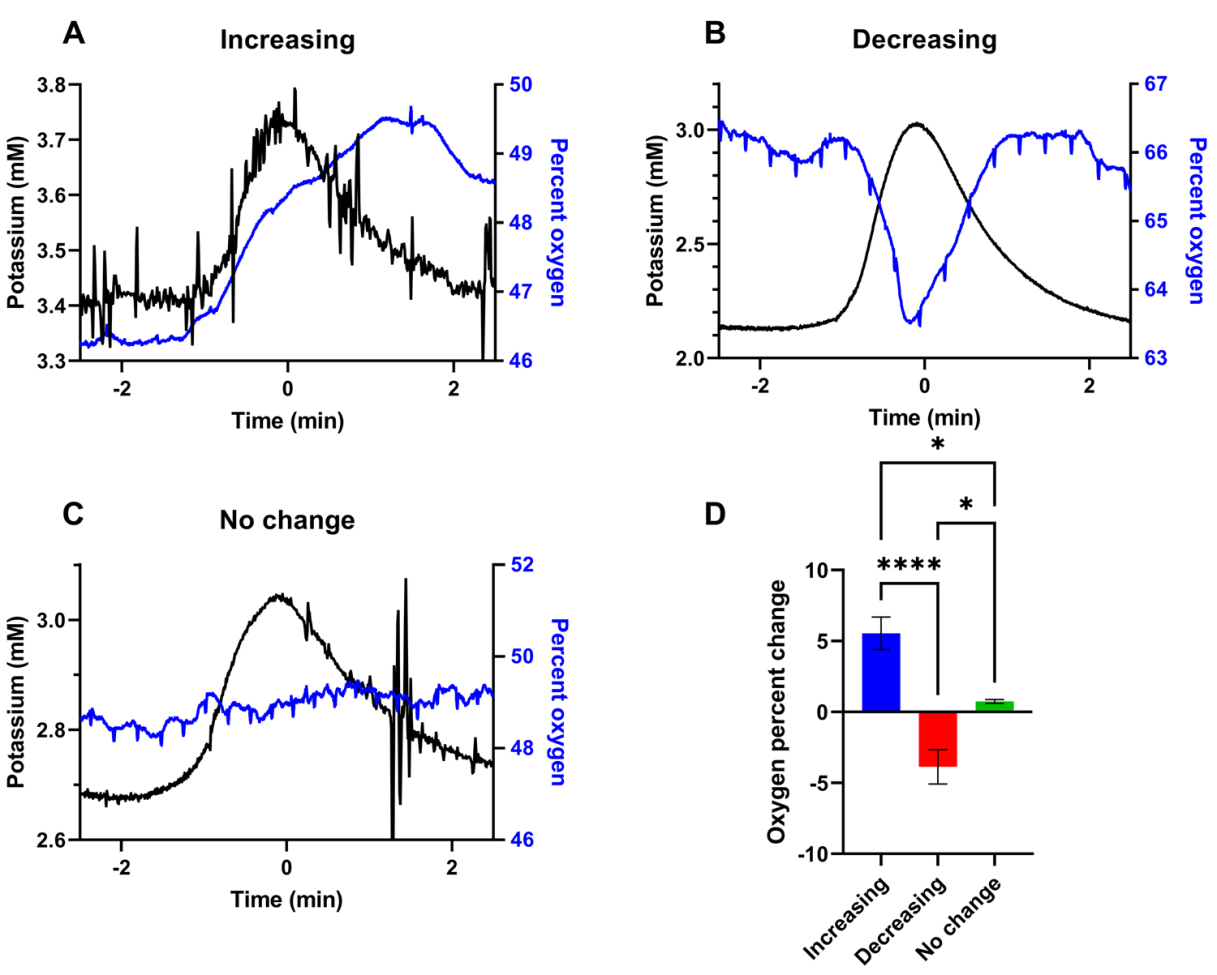
Mean of the K^+^ transients (black) evoked by needle pricks to the cortical surface and the corresponding mean of the O_2_ response (blue), grouped according to the classification of the O_2_ response: (A) O_2_ transient increases; (B) O_2_ transient decreases; (C) O_2_ null responses (*t* = 0 corresponds to the peak of the K^+^ transient.( D) The percent O_2_ changes between classifications are significantly different (* = *p* < 0.05, **** = *p* < 0.00005, one-way ANOVA, Tukey’s post hoc test).

Acute SD recordings were performed in *n* = 11 anesthetized rats. Starting 2 h after the completion of probe insertion, the brain surface was manually pricked with a hypodermic needle via a burr hole through the skull. Each rat was pricked 3 times with a 30 min interval between each prick. Of the 33 pricks administered, 19 evoked a K^+^ transient to indicate that SD occurred (Figure 2). Consistent with our prior report,^26^ some needle pricks failed to elicit a K^+^ transient (Figure S1 in the Supporting Information provides representative raw data from individual rats that responded to 3, 2, or 1 pin-prick): we assume that the prick did not evoke SD or that the evoked SD did not spread to the vicinity of the microdialysis probe.

During SD, dialysate O_2_ levels transiently increased, transiently decreased, or did not change (Figure 2). To objectively classify the O_2_ responses, we determined an O_2_ threshold of 3 times the standard deviation of the signal over the 2 min period just prior to the onset of the K^+^ transient. If the change in O_2_ at the peak time of the K^+^ transient exceeded this threshold, then the O_2_ response was classified as a transient; otherwise, it was classified as a null response. Accordingly, the 19 evoked SDs produced 7 O_2_ transient increases (Figure 2A), 7 O_2_ transient decreases (Figure 2B), and 5 O_2_ null responses (Figure 2C). The percent changes in O_2_ between response classifications were significantly different (Figure 2D).

The finding in Figure 2 that manually evoked SD produces a variety of O_2_ responses, including transient increases, transient decreases, and null responses, stands in slight contrast to the results of Varner et al.,^26^ who reported that manually evoked SDs produce transient decreases in dialysate glucose with only a few (15%) null responses: manually evoked SD did not cause transient increases in glucose. However, the outcome is different in the case CCI-evoked SD, as discussed further below.

**Figure 3:**
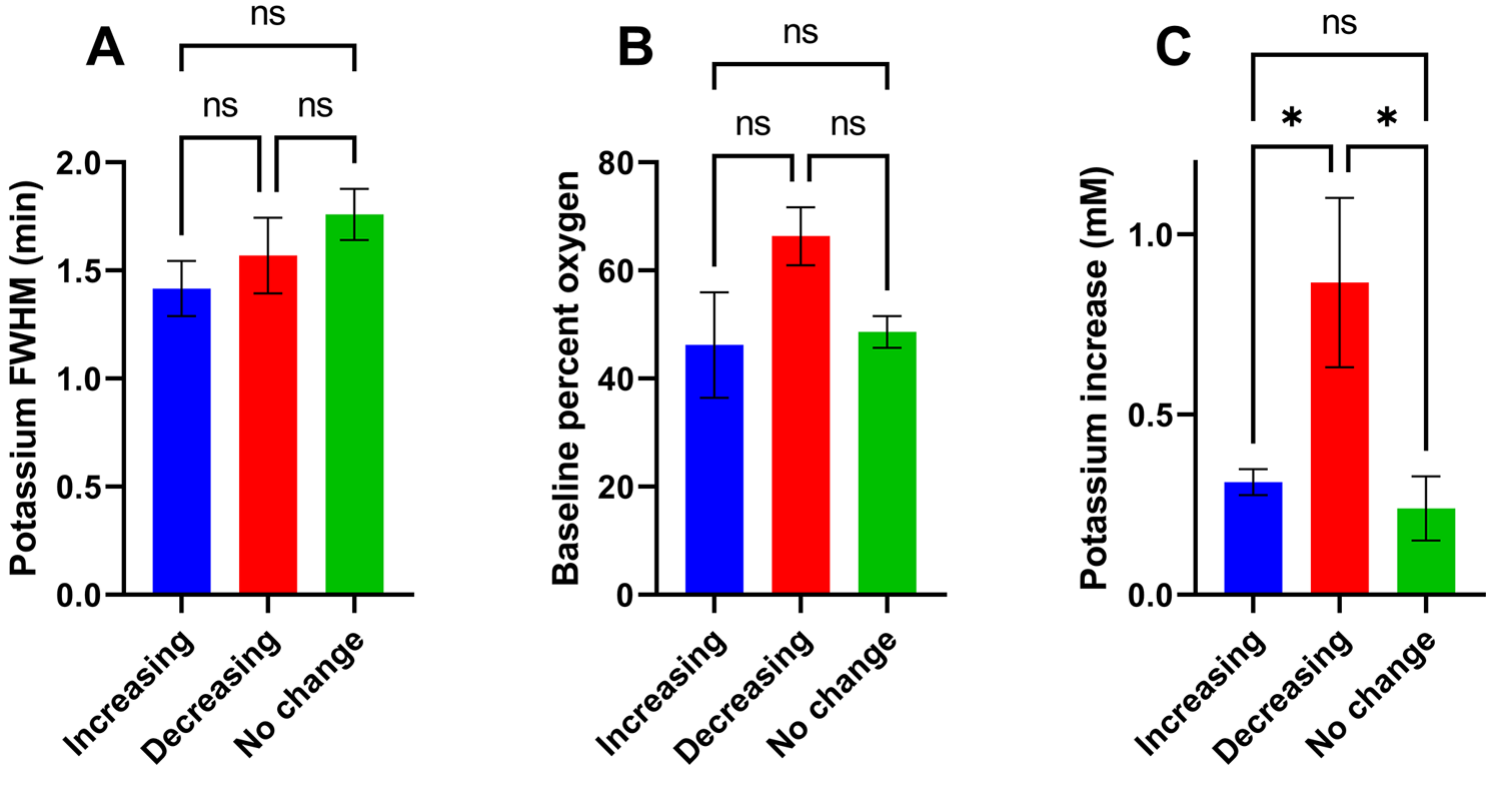
Mean ± SEM of three descriptive parameters of the manually evoked SD responses, according to the classification of the O_2_ response. (A) The full width at half-maximum of the K^+^ transient is not significantly different between classes. (B) The pre-SD O_2_ level is not significantly different between classes, although a trend toward increased pre-SD O_2_ is present in the case of the O_2_ transient decreases. (C) The amplitude of the K^+^ transient is significantly increased in the case of the O_2_ transient decreases (*p < 0.05, one-way ANOVA, Tukey’s post hoc test).

We quantitatively evaluated the responses in each classification (Figure 3). There are no significant differences between the durations of the K^+^ transients, quantified by their full width at half-maximum (Figure 3A). There are no significant differences between the baseline O_2_ levels prior to the onset of the K^+^ transient (Figure 3B), although baseline O_2_ trended higher prior to O_2_ transient decreases. There are, however, significant differences between the amplitudes of the K^+^ transients between classifications: the amplitudes of the K^+^ transients were significantly larger when accompanied by transient decreases in O_2_ (Figure 2B and Figure 3C). It is well established that tissue repolarization after SD demands large amounts of energy: we have previously reported that this energy demand drives a decrease in dialysate glucose^26,28,30−33^ and extend that finding here by documenting a concomitant transient decrease in dialysate O_2_. However, only the larger amplitude K^+^ transients are accompanied by transient decreases in dialysate O_2_.

Together, Figures 2 and 3 suggest that SDs might produce a range of metabolic consequences in brain tissue. SDs with low-amplitude K^+^ responses are accompanied by no O_2_ response or an increase in O_2_ that is possibly due to the known hyperemic response to SD. On the other hand, when the K^+^ amplitude is large, the SD is accompanied by a transient decrease in O_2_, suggesting that such SDs create a metabolic crisis because the O_2_ demand exceeds the vascular supply. During this work, however, the largest K^+^ amplitudes were observed during the probe insertion response (>3 mM, Figure 2), but these responses were not accompanied by any apparent decrease in O_2_; thus, it appears that the metabolic consequences of probe insertion and a pin-prick are different.

The literature most often describes SD as an “all or nothing” mass depolarization event,^16^ wherein brain tissue either depolarizes or does not. The finding of variations in the amplitude of SD responses (Figures 2 and 3) is not in strict agreement with this “all or nothing” model. As discussed in the Supporting Information, variations in the amplitude of SD responses have also been observed in prior microdialysis studies from the Boutelle group (Figure S2).^37^ On the other hand, Marinesco and colleagues, who monitored brain glucose with parenchymal microbiosensors, observed a consistent, and deeper, decrease in glucose during SD, more consistent with the “all or nothing” model.^38−40^ We speculate that the size and geometry of the recording devices play a role in these contrasts. The microbiosensors, which have tips 100 μm in length and 40 μm in diameter, perform spatially localized measurements. This increases the chances that the entire tip senses the SD response. Microdialysis probes are considerably larger (herein, 4 mm in length and 280 μm in diameter) and perform spatially averaged measurements. This increases the chances that only a portion of the probe monitors the SD response, leading to variations in the response amplitude due to averaging.

Overall, the performance differences between microdialysis probes and implanted microsensors are well-known. Microsensors deliver high spatial and temporal resolution; however, they are less suitable for chronic measurements and are not approved for clinical use. Microdialysis probes deliver less spatial and temporal resolution but rather perform a spatially averaged measurement. On the other hand, they monitor over a larger region of tissue, which might increase the chances of detecting more SD events. Microdialysis is suitable for chronic measurements lasting several days and is approved for clinical use.

### Chronic 7-Day Measurements: SD after CCI

**Figure 4:**
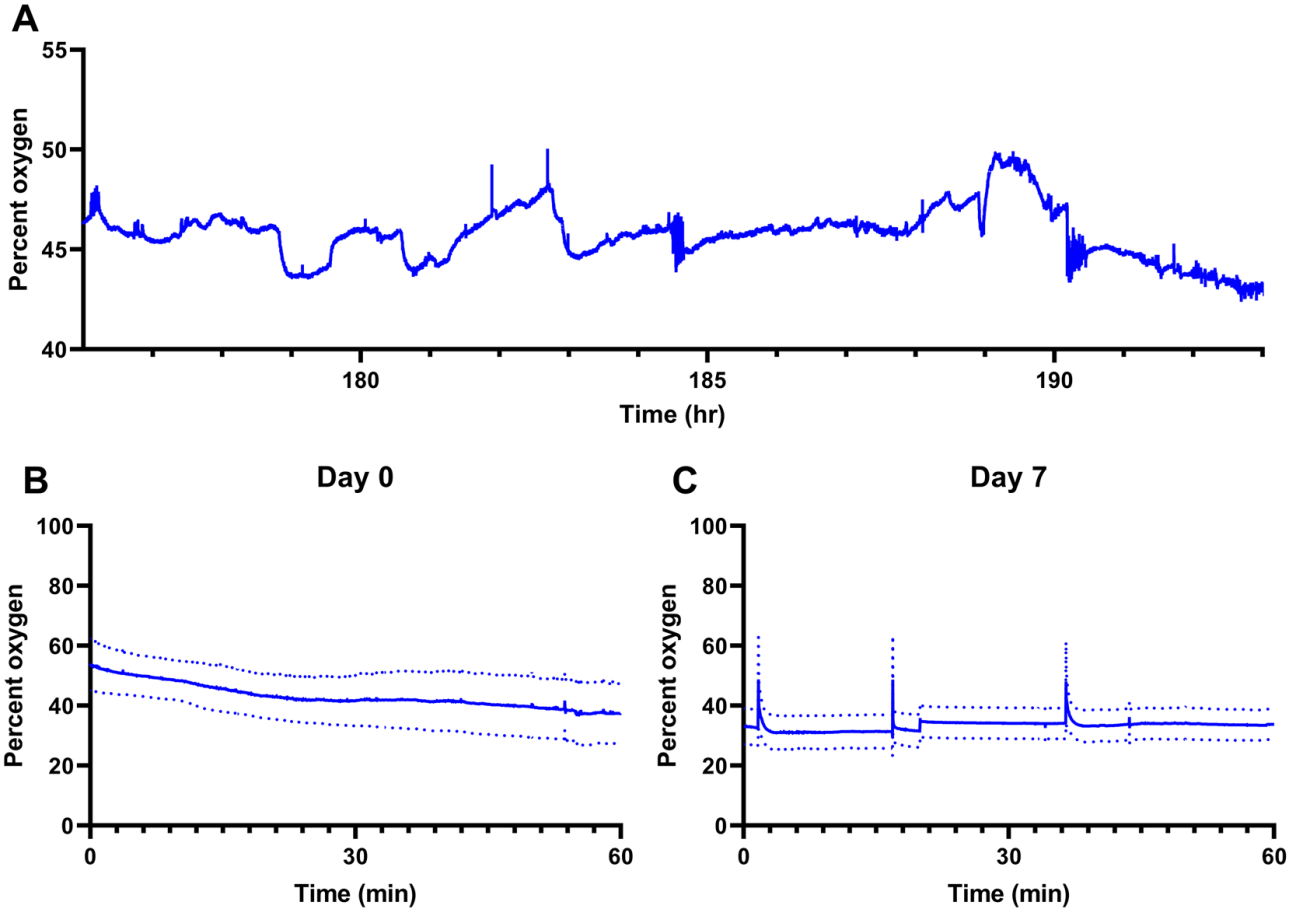
Dex-enhanced coMD monitoring of O_2_ in the cortex of control rats (no CCI injury) on days 0 and 7 following probe insertion. (A) Trace of O_2_ in dialysate from an individual rat on day 7. (B, C) Mean O_2_ from *n* = 4 rats on days 0 (B) and 7 (C) after probe insertion (mean ± SEM).

A group of rats (*n* = 6) was anesthetized, received a CCI, and had a microdialysis probe inserted 3 mm anterior to the injury site. Five of these animals underwent 7 days of Dex-enhanced coMD with the apparatus depicted in Figure 1C. One animal died on the day following the CCI and probe insertion without exhibiting SD. One other rat exhibited no post-CCI SDs. The other 4 injured rats exhibited a total of 63 SDs, ranging from 3 to 26 per rat. After CCI, all 6 rats exhibited a long-term decline in dialysate O_2_ to near-zero levels. A group of control rats (*n* = 4) received a microdialysis probe but neither a CCI nor a sham craniotomy. These noninjured control rats exbited neither SD nor long-term changes in O_2_ or K^+^ over the 7 days following probe insertion. Figure 4A shows a representative O_2_ trace from a control animal on day 7 postinsertion. Dialyate O_2_ levels hover around 45% with fluctuations around 5% of unknown origin. Figure 4B and Figure 4C show mean O_2_ measured in 60 min time windows in the control rats on days 0 (the day of insertion) and 7, respectively, following probe insertion. The mean O_2_ levels were 38.0 ± 10.0 on day 0 and 33.8 ± 5.1 on day 7; these values are not significantly different.

Figure 4 shows baseline O_2_ level near 40%, where 100% corresponds to aCSF equilibrated with O_2_ and 0% corresponds to aCSF equilibrated with nitrogen (see Figure 1). Thus, 40% O_2_ corresponds to ∼300 mmHg, which is about 10-fold higher than the usual value of PbtO_2_, 20–30 mmHg.^14,41^ Our study was not designed to investigate this discrepancy, but our use of Dex-retrodialysis to mitigate gliosis and vascular disruption at microdialysis probe tracks is a possible contributor. It is also the case that the microdialysis perfusion fluids used during this work were equilibrated with the ambient lab air; i.e., they contained a source of O_2_ that is not present when PbtO_2_ is measured with conventional electrochemical devices.

**Figure 5:**
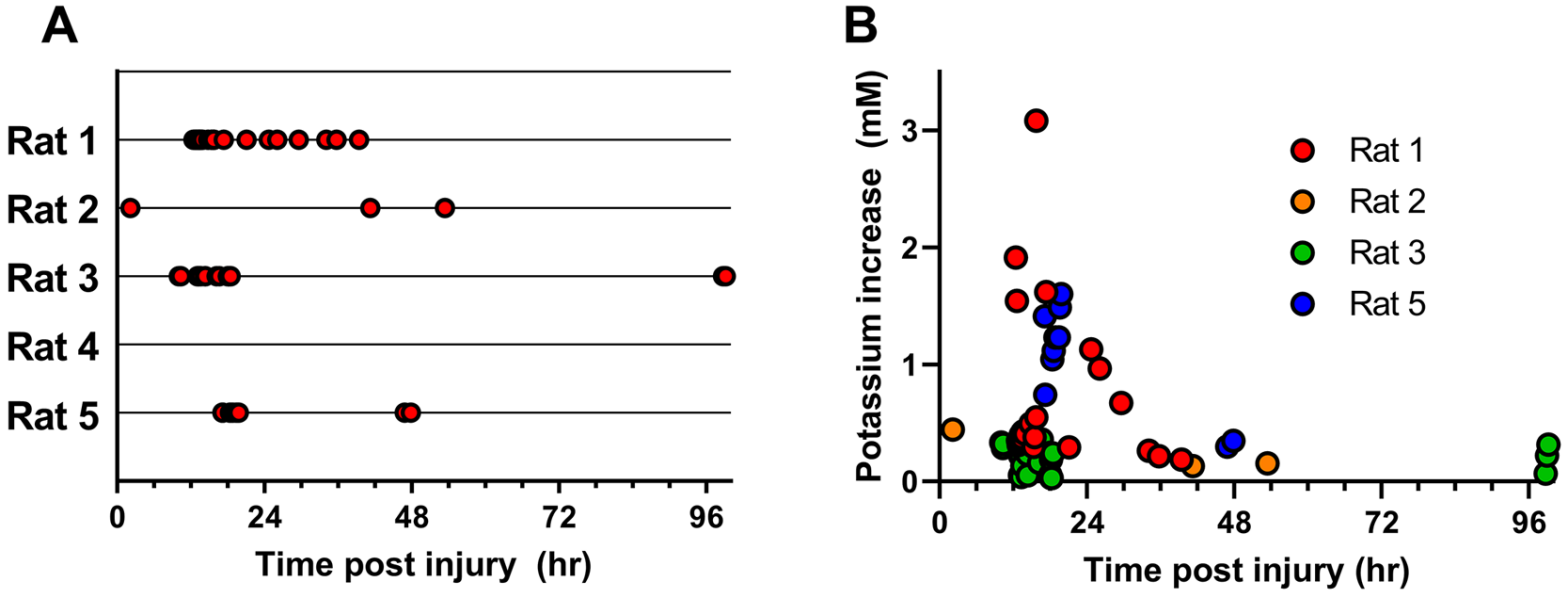
(A) Distribution of post-CCI SDs across the 5 rats monitored for 7 days. All SDs occurred within the first 5 days, with the majority occurring within the first 48 h. (B) Amplitude of the K^+^ transients observed over time. Numerous transients with amplitudes below 1 mM occurred during the first 5 days. Several transients with amplitudes between 1 and 3 mM occurred between 12 and 30 h post-CCI.

Figure 5A shows the temporal distribution of SD events in 5 injured rats. All SD events occurred within 5 days of CCI, with the majority (59 of 63) occurring within the first 48 h but only one during the first 8 h. Thus, 8–48 h after CCI appears to be a critical SD window. This SD window is in good agreement with existing literature. In juvenile rats, for example, oxidative metabolism of glucose begins to fail around 6 h following injury,^42^ possibly indicating that poor energy availability to the Na^+^/K^+^ pumps that maintain cell membrane ion gradients could potentially be the inciting event that triggers SD activity. Caspase-3 immunoreactivity reaches a maximum in cortex at 48 h after CCI,^43^ possibly indicating that apoptosis is beginning to clear the most distressed cells responsible for SD initiation and propagation.

As with manually evoked SD (Figure 3), SD post-CCI exhibited K^+^ transients with a range of amplitudes. K^+^ transients with amplitudes below 1 mM occurred over the entire 5-day interval after CCI (Figure 5B). However, K^+^ transients with notably larger amplitudes of 1–3 mM occurred 12–30 h after CCI (Figure 5B). The data points in Figure 5B are color-coded to show points from individual rats. Some rats showed more variation in K^+^ amplitude than others, although the exact reason for this is unclear. As mentioned above, given the usual depiction of SD as an “all or nothing” event, the variation in K^+^ transient amplitudes noticed here may be reflective of the volume of tissue impacted by the SD rather than the local SD intensity. This, however, is potentially valuable information, indicating that larger volumes of brain tissue are more susceptible to SD within certain time windows after injury: it remains to be seen how these data from the CCI model would translate to the case of TBI in patients.

**Figure 6:**
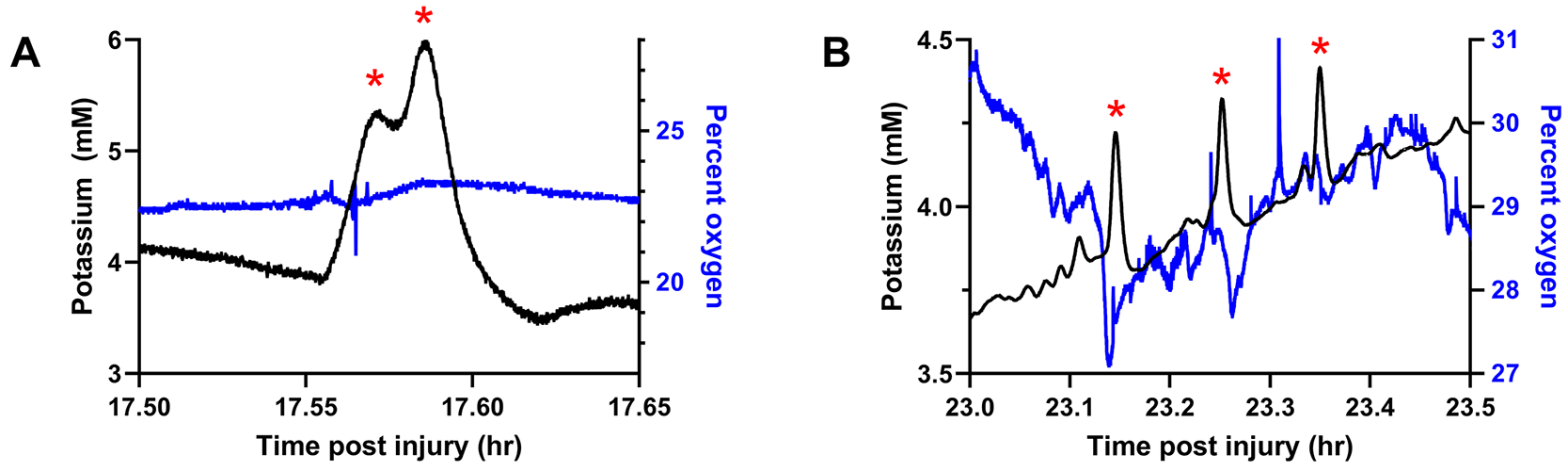
Dialysate O_2_ (blue) and K^+^ levels (black) were monitored for 7 days post-CCI. (A) An example of two SDs in quick succession with a null O_2_ response. (B) An example of three SDs occurring at ∼6 min intervals: the first two SDs are accompanied by transient decreases in O_2_, but the third is not. Red asterisks mark SDs.

SD after CCI produced a range of O_2_ responses. However, due to the fact that many SDs appeared in quick succession (so-called SD clusters^44,45^) and because dialysate O_2_ sometimes exhibited baseline changes (discussed further in the next section), objectively classifying these O_2_ responses was not practical. Figure 6 shows some representative responses. Figure 6A shows an example of two SDs occurring in quick succession, with the second K^+^ transient beginning before the first one finished: such SD clusters have been previously associated with progressive declines in glucose.^32^ However, in this example, no O_2_ response was evident (null response). Figure 6B shows three K^+^ transients occurring about 6 min apart: the first two produced transient O_2_ decreases but the third did not. Unlike the case of manually evoked SD (Figure 3), SDs after CCI were accompanied by null O_2_ responses (Figure 6A) and transient O_2_ decreases (Figure 6B) but not transient O_2_ increases.

### Chronic 7-Day Measurements: Long-Term Events after CCI

**Figure 7:**
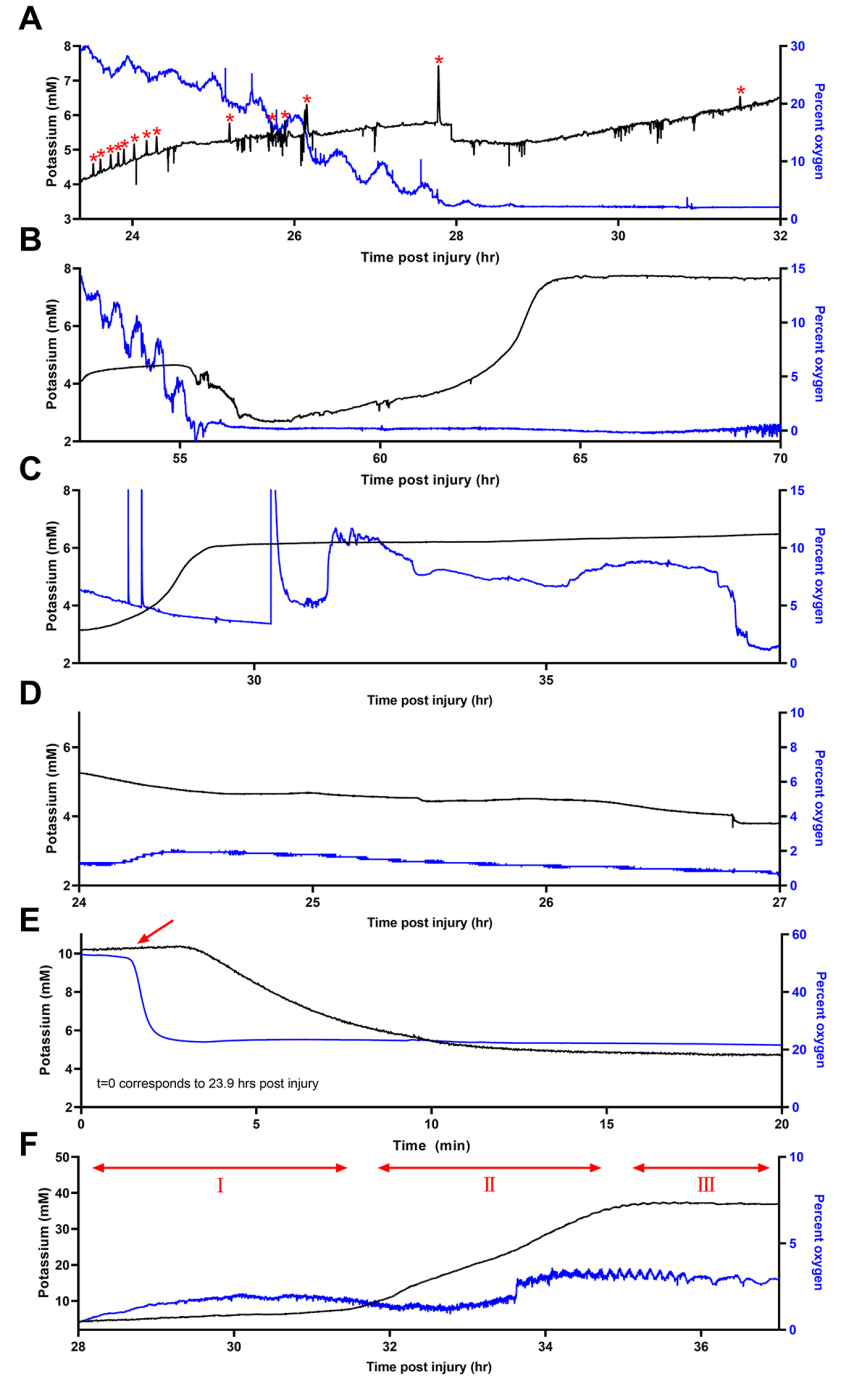
All 6 rats that received a CCI exhibited near-zero dialysate O_2_ levels postinjury. Multiple post-CCI SDs (red asterisks) and associated O_2_ fluctuations were detected in 4 of these rats. Additionally, long-term declines in O_2_ and increases in K^+^ occurred post-CCI in (A) rat 1, (B) rat 2, and (C) rat 3. In two animals, monitoring did not capture the decline itself, but we observed the low O_2_ and elevated K^+^ levels in (D) rat 4 and (E) rat 5. In the first few minutes of (E), a calibration solution was replaced with brain dialysate (red arrow): the O_2_ level dropped, showing that the brain tissue surrounding the probe extracts O_2_ from the perfusion fluid. (F) In rat 6, which died on the day after CCI, recordings of O_2_ were persistently low, while K^+^ was near normal initially, rose slowly in region I, rose rapidly in region II, and then plateaued near 40 mM in region III and remained elevated until death occurred.

In all 6 rats monitored after CCI, baseline dialysate O_2_ fell to levels too low to measure. In 5 of these rats, baseline O_2_ levels began at near-normal levels and, at some point, progressively declined to near-zero (Figure 7A–E). Once this decline occurred, O_2_ did not recover. These experiments were terminated 7 days after CCI because by that time all injured rats had exhibited the decline. We emphasize that the decline in O_2_ was not due to any electrode failure: the electrochemical O_2_ signal remained too low to measure even with freshly refurbished and recalibrated electrodes. In some cases, as in Figure 7A–C, as O_2_ declined, K^+^ steadily increased and K^+^ transients indicating SD were observed in rat 1 after the O_2_ decline (Figure 7A); such observations confirm that the membrane of the microdialysis probe is continuing to exchange substances with the extracellular space. Furthermore, dialysate O_2_ levels fell below the O_2_ level from the air-equilibrated aCSF delivered to the probe inlet. The data in Figure 7E were recorded during and immediately following a calibration of the K^+^ electrode. As the last of the air-equilibrated calibration solution was replaced by brain dialysate in the first several minutes of Figure 7E, O_2_ dropped from above 50% to approximately 20%, indicating that O_2_ was being lost from the perfusion fluid to the surrounding tissue, i.e., that the surrounding tissue was extracting O_2_ from the probe, confirming that the microdialysis membrane retains its permeability to O_2_. Whether the O_2_ is consumed by nearby cells or is carried away by the circulatory system^46^ is unknown at this time. These observations confirm that the decline of dialysate O_2_ is not attributable to a loss in the permeability (i.e., blockage) of the dialysis membrane.

As mentioned above, one rat died on the day after CCI. Dialysate O_2_ in this individual was persistently low throughout the monitoring period (Figure 7F). Starting at 28 h post-CCI (region 1 of the plot), K^+^ levels began a persistent rise. In region II, K^+^ rose more rapidly. In region III, K^+^ levels plateaued near 40 mM, far higher than we have observed in any other case, and remained at that high level until death occurred.

The long-term declines in dialysate O_2_ reported here show a similarity to the long-term declines in glucose reported in our previous work.^32^ In several cases, we also observed in similar instances that dialysate K^+^ levels increased as dialysate glucose levels declined. Like the O_2_ declines reported here, the glucose declines were also essentially permanent: once glucose declined, it did not recover. As with the O_2_ declines reported here, there was no evidence that the glucose declines were associated with any loss in permeability of the microdialysis membrane.^33^

## Conclusion

Our findings show that Dex-enhanced coMD offers the technical ability to monitor dialysate O_2_ levels both acutely and over the longer term (7 days) with a singly implanted microdialysis probe. Dex-enhanced coMD provided new information on how O_2_ levels respond to acute, manually evoked SD as well as SD after CCI. Dex-enhanced coMD also documents long-term changes in tissue O_2_, specifically a long-term and apparently permanent decline after CCI. We conclude that the decline is a marker of metabolic abnormality arising after CCI injury.

Microdialysis has been used to monitor brain potassium, sodium, glucose, lactate, glutamate, and more after TBI.^27,30,47−50^ Thus, microdialysis has the potentially significant ability to monitor both the ionic disruptions that indicate SD and additional small molecules that serve as markers for the metabolic impact of SD. However, a glial barrier forms at the probe track over a few days after implantation.^22,24,25^ This barrier interferes with long-term monitoring by degrading the performance of microdialysis.^51^ Dex retrodialysis resolves this issue by preventing gliosis, preserving blood flow, and preserving glia and neurons in the vicinity of the probe tracks for at least 10 days after probe implantation.^26,33,52^ This study is the first attempt to deploy Dex-enhanced brain microdialysis for O_2_ monitoring.

Our findings reveal that SD induces a variety of O_2_ responses, which is similar to our previous observations on glucose.^26,32^ However, SDs induced with pin-pricks did not induce transient increases in glucose. Only those SDs exhibiting large-amplitude K^+^ transients were accompanied by transient O_2_ decreases. The longer-term measurements after CCI revealed a slow decline in O_2_, sometimes coincident with a slow increase in K^+^. These observations bear a striking resemblance to those of glucose.^32^ Chronically elevated K^+^ may contribute to lesion expansion in secondary injury^53^ or may be indicative of blood– brain barrier disruption.^54,55^

Several prior studies suggest that cerebral glucose utilization shifts from oxidative phosphorylation to less efficient anaerobic pathways after brain injury.^56,57^ This less efficient glucose utilization is a potential explanation for the CCI glucose decline after CCI. The data in Figure 7 raise the possibility that an insufficiency in O_2_ availability might drive this shift.

While SDs are clinically monitored by electrocorticography,^58,59^ SDs cause many chemical changes that are valuable to measure, including the changes in O_2_ levels reported here.^60,61^ Reduced brain tissue oxygenation in TBI patients is associated with negative outcomes,^13,62^ and there is evidence that care directed by brain oxygenation may be associated with improved outcome when compared to conventional ICP or cerebral perfusion pressure-managed care.^14^ Brain O_2_ monitoring can be accomplished in many ways, including with fast scan cyclic voltammetry^61,63^ and clinically with implanted polyethylene-coated platinum electrodes.^64,65^ Oxygen measurements using microdialysis in human patients is currently not presently possible because the commercial probes approved for clinical use have polyurethane connecting lines, which are highly O_2_-permeable. However, our data emphasize the potential importance of coupling chemical sensing to SD monitoring in the injured brain.

## Methods

### Reagents and Solutions

Artificial cerebrospinal fluid (aCSF) contained 142.0 mM NaCl, 1.2 mM CaCl_2_, 2.7 mM KCl, 1.0 mM MgCl_2_, and 2.0 mM NaH_2_PO_4_ (reagents from Sigma-Aldrich) and was adjusted to a pH of 7.4. 10 μM and 2 μM solutions of dexamethasone sodium phosphate (APP Fresenius Kabi USA LLC, Lake Zurich, IL) were prepared in aCSF. Microdialysis perfusion fluids were filtered with sterile Nalgene filters (PES, 0.2 μm pore size; Fisher, Pittsburgh, PA) and equilibrated with ambient air before use. Solutions were prepared with ultrapure water (Nanopure; Barnstead, Dubuque, IA).

### Microdialysis

Microdialysis probes were constructed in-house using 280 μm OD regenerated cellulose hollow fiber membranes cut to a length of 4 mm (18 kDa molecular weight cutoff; SpectraPor RC, Spectrum, Rancho Domingues, CA). Inlet and outlet lines were made from fused silica capillaries (75 μm ID, 150 μm OD; Polymicro Technologies, Phoenix, AZ). Probes were flushed with and soaked in 70% ethanol, then flushed with 10 μM dexamethasone (Dex) in aCSF prior to insertion into the rat brain. Probes were inserted into the brain using a carrier arm of a Kopf stereotaxic frame: the manual insertion procedure lasted approximately 4 min. The probes were perfused with air-equilibrated fluid by means of gastight syringes driven by a syringe pump (Harvard Apparatus, Holliston, MA) at a flow rate of 1.67 μL/min. The probes were perfused with 10 μM Dex for the first 24 h, then with 2 μM DEX for 4 days, and then without Dex for the remainder of the experiment.

### Acute Surgical Procedures

Animal procedures were approved by the University of Pittsburgh’s Institutional Animal Care and Use Committee. Male Sprague-Dawley rats (250–350 g; Charles River, Raleigh, NC) were anesthetized with isoflurane (5% induction, 2.5% maintenance by volume O_2_) and placed in a stereotaxic frame. Rats in the acute group (*n* = 11) remained under anesthesia for the entire experiment. The microdialysis probe was inserted into the cortex at the following coordinates: 4.2 mm posterior to bregma, 1.5 mm lateral to midline, and 4 mm below dura at a 51° angle to the vertical plane; all measurements were made using flat skull. With this insertion procedure, the entire 4 mm active portion of the probe was placed in the cortex. A second hole was drilled through the skull 4.5 mm anterior to the probe on the ipsilateral side. After 2 h, an 18 G hypodermic needle was used to manually prick the surface of the cortex through the second hole. Three pin pricks were performed per animal, with a 30 min waiting period in between.

### Chronic Surgical Procedures

For animals monitored for 7 days, a Leica Impact One (Leica Biosystems, Buffalo Grove, IL) was used to create a model of mild to moderate TBI. A craniotomy was performed on *n* = 6 rats posterior to bregma on the right hemisphere, and the exposed dura was struck at a velocity to 4.00 m/s, with a 100 ms dwell time to a depth of 2.2 mm. The probe was then inserted 3.0 mm anterior to the craniotomy, again at a 51° angle to ensure the entire probe was placed in the cortex. The probe was secured with bone screws and acrylic cement, and the incision was closed with sterile sutures. Probes were also inserted into the cortex of control rats (*n* = 4); control rats did not receive a CCI injury. These rats were housed in a Raturn microdialysis bowl (MD-1404, BASI, West Lafayette, IN) for the 7 days of microdialysis.

### Oxygen and Potassium Monitoring

The outlet of the probe was connected to a 3D printed microfluidic chip; the design is described in detail elsewhere.^30^ Electrodes for detecting O_2_ and K^+^ were inserted into the flow stream of the chip (200 μm diameter) using 3D printed holders that screw into place without tools. The O_2_ sensor was a needle-style electrode fabricated by threading 50 μm diameter platinum and silver wires (Goodfellow, Huntingdon, U.K.) through a 27 G needle and sealing them in place with Spurr low-viscosity epoxy (Sigma-Aldrich). The stainless-steel needle body was used as the counter electrode. K^+^ μISEs were constructed by casting a potassium-selective membrane (2 mg of potassium ionophore, 0.2 mg of potassium tetrakis(4-chlorophenyl)borate, 150.0 mg of bis(2-ethylhexyl) sebacate, and 66 mg of poly(vinyl chloride) dissolved in 1 mL of tetrahydrofuran; reagents from Sigma-Aldrich) into a fused silica capillary (Polymicro Technologies, Phoenix, AZ). The electrode was backfilled with aCSF, and an Ag/AgCl internal reference electrode was inserted (50 μm diameter; Goodfellow). An additional needle-style Ag/AgCl external reference for the potassium electrode was also inserted into the microfluidic chip. All data were time corrected to account for the transit time between the electrodes in the microfluidic chip.

### Data Acquisition, Electrode Calibration, and Analysis

In the acute needle prick experiments, K^+^ data were collected using homemade printed circuit boards courtesy of the University of Pittsburgh Electronics Shop connected to a PowerLab 2/26 data acquisition system using LabChart 8 software (AD Instruments, Colorado Springs, CO). O_2_ data were acquired using a CHI 1205C potentiostat (CHInstruments, Austin, TX) by holding the Pt working electrode at −0.6 V vs Ag/AgCl. During chronic experiments, an additional custom potentiostat was constructed by the University of Pittsburgh Electronics Shop connected to a second channel of the PowerLab 2/26. Oxygen was reduced at the Pt working electrode by holding the potential at −0.6 V vs Ag/AgCl. Calibration was performed using a set of LabSmith SPS-01 syringe pumps fitted with gastight syringes and 3-port selector valves (LabSmith, Inc., Livermore, CA). All connections were made with fused silica tubing and gastight connectors. Data analysis was conducted using MATLAB (Mathworks Inc.), and statistical analysis was done in GraphPad Prism 9.0 (GraphPad Software, San Diego, CA).

## Supporting Information Available

Figure S1 showing data from study of SDs and Figure S2 showing reanalysis of data (PDF)

## Abbreviations

TBI: traumatic brain injury
CCI: controlled cortical impact
SD: spreading depolarization
Dex: dexamethasone
coMD: continuous online microdialysis
aCSF: artificial cerebrospinal fluid
μISE: microion-selective electrode
