# Supplementary material for: Dexamethasone-Enhanced Continuous Online Microdialysis for Neuromonitoring of O2 after Brain Injury
Source: ACS Chem Neurosci. 2023 Jun 27;14(14):2476–86. doi: 10.1021/acschemneuro.2c00703 (PMC10360069; doi:10.1021/acschemneuro.2c00703)
Supplement: Supplementary file 1 — cn2c00703_si_001.pdf [file cn2c00703_si_001.pdf]

Supplementary Information

Dexamethasone-Enhanced Continuous Online Microdialysis for Neuromonitoring of O_2 after Brain Injury

Elaine M. Robbins[1], David O. Okonkwo[2], Martyn G. Boutelle[3], Adrian C. Michael[1*]

[1] Department of Chemistry University of Pittsburgh, Department of Chemistry University of Pittsburgh, 219 Parkman Ave Pittsburgh, PA 15260, USA
Phone: 412 624-8560

[2] Department of Neurological Surgery, University of Pittsburgh School of Medicine, Pittsburgh, PA, 15213, USA

[3] Department of Bioengineering, Imperial College London, London SW7 2AZ, United Kingdom

Figure S1 provides raw data from three individual rats from the acute study of SDs. The top trace is from a rat that responded to 3-of-3 pin-pricks, the middle trace is from a rat that responded to 2-of-3 pin pricks, and the bottom trace is from a rat that responded to 1-of three pin pricks. Note that the responses are delayed with respect to the time of the pin-prick: this is the transit time of the dialysate sample through the outlet tubing of the probe.

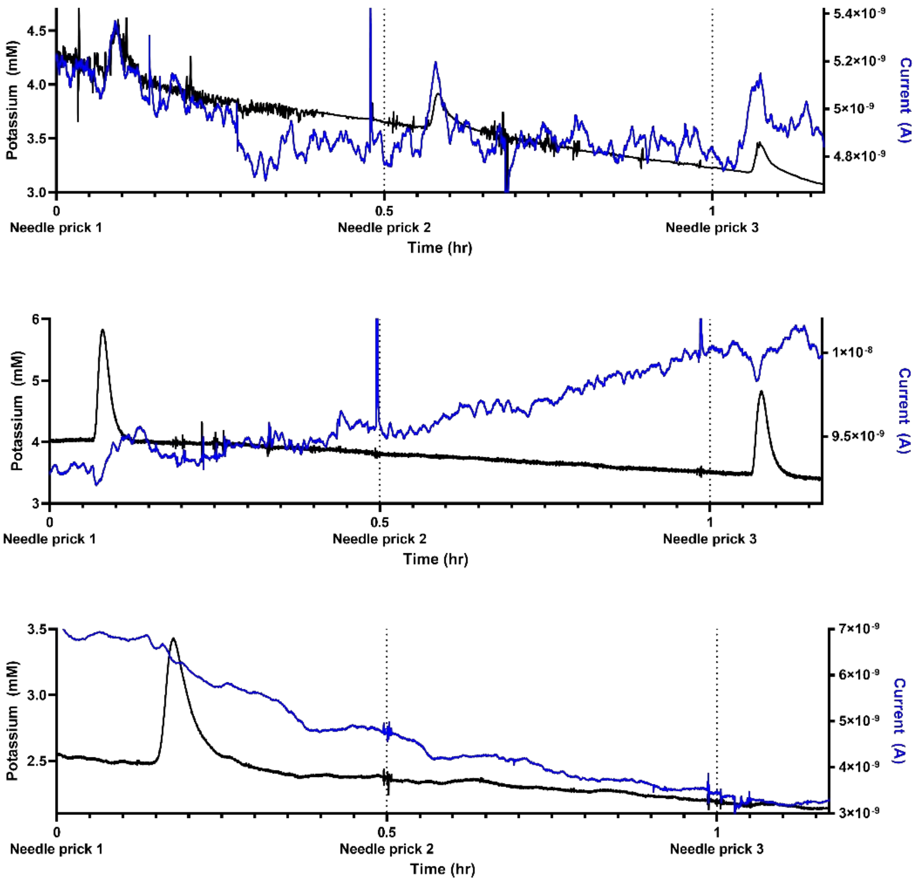

Figure S2 presents a reanalysis of some data previously published in the report of Rogers et al (2013).[1] Rogers et al used continuous-online microdialysis to record K^+ and glucose responses to spreading depolarization induced by pin-pricks to the cortical surface of rats anesthetized with isoflurane (the methods and procedures of the prior and present studies are similar but not identical: please refer to the prior report for details). Similar to the observations in Figure 2 and 3 of the main text, Rogers et al observed a variation in the amplitudes of the K+ and glucose transients associated with SD (Supplementary Figure 1). Moreover, the K^+ and glucose amplitudes were correlated with each other, i.e. larger increases in K^+ were correlated with larger decreases in glucose.

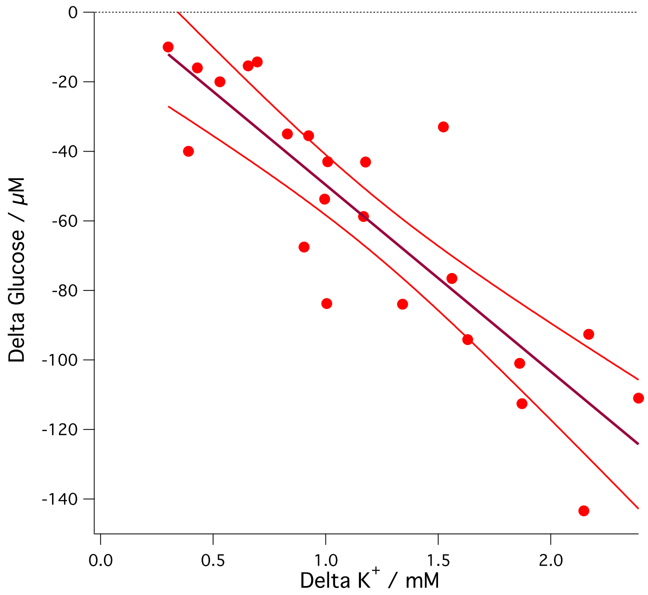

REFERENCE

1. Rogers, M.L.; Feuerstein, D.; Leng Leong, A.; Takagaki, M.; Niu, X.; Graf, R.; Boutelle M.G. Dynamic Neurometabolic Changes during Spreading Depolarization. *ACS Chem. Neurosci.,* **2013**, 4, 799-807. dx.doi.org/10.1021/cn400047x
